# Hospital choice in Germany from the patient’s perspective: a cross-sectional study

**DOI:** 10.1186/s12913-017-2712-3

**Published:** 2017-11-13

**Authors:** Werner de Cruppé, Max Geraedts

**Affiliations:** 1 Institute for Health Systems Research, School of Medicine, Faculty of Health, Witten/Herdecke University, Alfred-Herrhausen-Strasse 50, 58448 Witten, Germany; 2 Institute for Health Services Research and Clinical Epidemiology, Philipps-Universität Marburg, Karl-von-Frisch-Strasse 4, 35043 Marburg, Germany

**Keywords:** Hospital choice, Decision-making, Patient autonomy, Hospital, Hospitalisation, Germany, Cross-sectional study

## Abstract

**Background:**

In many countries health policy encourages patients to choose their hospital, preferably by considering information of performance reports. Previous studies on hospital choice mainly have focused on patients undergoing elective surgery. This study examined a representative sample of hospital inpatients across disciplines and treatment interventions in Germany. Its research questions were: How many patients decide where to go for hospital treatment? How much time do patients have before admission? Which sources of information do they use, and which criteria are relevant to their decision?

**Methods:**

Cross-sectional observational study covering 1925 inpatients of 46 departments at 17 hospitals in 2012. The stratified survey comprised 11 medical disciplines (internal medicine, gynaecology, obstetrics, paediatrics, psychiatry, orthopaedics, neurology, urology, ENT and geriatrics) on 3 hospital care levels representing 91.9% of all hospital admissions to inpatient care in Germany in 2012. The statistical analysis calculated the frequency distributions and 95% confidence intervals of characteristics related to the hospital choice.

**Results:**

63.0% [60.9–65.2] of patients in Germany chose the hospital themselves, but only 21.1% [19.3–22.9] had more than one week to decide prior to admission. Major sources of information were personal knowledge of hospitals, relatives, outpatient health professionals and the Internet. Main criteria for the decision were personal experience with a hospital, recommendations from relatives and providers of outpatient services, a hospital’s reputation and distance from home. Specific quality information as provided by performance reports were of secondary importance.

**Conclusions:**

A majority of patients in the German health system choose their hospital freely. Providers of outpatient health care can have an important “agent” function in the quality-oriented hospital choice especially for patients with little time prior to admission and those who do not decide themselves. Hospitals have an impact on patients’ future hospital choices by the treatment experience they provide to patients.

**Electronic supplementary material:**

The online version of this article (10.1186/s12913-017-2712-3) contains supplementary material, which is available to authorized users.

## Background

Free choice of healthcare providers, especially of hospitals, is a declared health policy objective in many countries and in Germany as well [1–6]. The reasons to promote free provider choice are diverse. In market-oriented health systems, like in the USA, free choice of healthcare providers is considered a competitive mechanism that improves quality and reduces costs [3, 4, 7]. Tax-funded and social security health systems expect a steering effect on the number of healthcare providers and the range of services offered, ultimately reducing waiting times and improving outcomes. [8–11]. Across health care systems, the free choice of healthcare providers constitutes an important element of patient autonomy. A more active role of patients in treatment processes is intended to improve therapy compliance and thereby outcomes [12–15], and promotes patient orientation as an independent quality dimension in health care [16, 17].

In Germany approximately 2000 hospitals with some 500,000 beds provide hospital care [rehabilitation clinics not included] with the hospitalist model as the standard provision of medical care for inpatients. They treat about 19 million inpatient cases with statutory and private insurance coverage per year [18]. About 88% of the 81 million inhabitants in Germany have statutory health insurance, and 11% are privately insured. A guideline of the Federal Joint Committee stipulates the hospital admission procedure for inpatients [19]. The Federal Joint Committee is the highest decision-making body of the joint self-government of physicians, dentists, hospitals and health insurance funds in the German health care system. Its respective guideline states that the indication for hospital treatment comes from outpatient doctors and is confirmed by the hospital physicians upon admission. The referring physician specifies “the two nearest reachable suitable hospitals …in appropriate cases” on the referral order [19]. In practice, however, patients are free in their choice of a hospital, encouraged by guides on patient rights as published by the Ministry of Health. Furthermore, in Germany patients are not registered with a specific hospital in their community, nor are primary care practitioners [54,000 in 2015] or specialised physicians in the ambulatory setting [94,000 in 2015] obliged to transfer a patient to a specific hospital. In Germany outpatient care is still provided mainly by medical specialists in their own practice in the ambulatory care setting. That is the reason for the hospitalist being the standard care model in hospitals. Thus the ambulatory care physician, who indicates hospital admission, is not an agent on behalf of a hospital. Some integrated care models provide a tight connexion with a specific hospital, especially with hospital owned policlinics, but patients are free to inscribe in such models with mostly a 12-month term of notice. Until now, though, very few patients are under these terms. For the sickness funds a free choice of hospitals does hardly imply different costs since diagnosis related groups were introduced in 2004 as reimbursement scheme for hospital treatments all over Germany with only minor differences between the 16 Laender. But it might occur that a patient is required to pay the transport to a distant hospital.

Informed decision is considered a core requirement for the free choice of healthcare providers. For this purpose, information on hospitals needs to be publicly available and known, easily accessible, comparable, structured and standardised. As to content, it should comprise scientifically validated and relevant quality indicators and be easily understandable in terms of language, scope and level of aggregation [20–25]. In Germany all hospitals have to publish obligatory quality reports whose structure and format is defined by the Federal Joint Committee. The reports are supposed to serve patients’ informed decision making when choosing a hospital and are freely available via Internet [26]. Numerous studies have analysed the significance of such performance information for patients and find it rather limited [27–32]. Although patient surveys have revealed much interest in quality information, it plays a minor role in the decision-making process even for elective interventions, and even if patients know and understand the quality information. Faber and colleagues [31] conclude that patient behaviour in this respect does not correspond to the model of market-oriented consumer choice, and Marshall and McLoughlin refer to the knowledge construction model supported by psychological and sociological studies as more relevant for patients’ decisions making in the healthcare context [33].

Studies on hospital choice have focused so far especially on two aspects: first, which criteria are important in the choice of a hospital from the patient perspective, and second, which sources of information do patients use. Numerous studies explore these two aspects [9, 22, 27–51], using various populations in qualitative, quantitative or experimental study designs. The selection lists for decision criteria and sources of information differ in terms of numbers, content, classification and assessment scale. A validated survey instrument for hospital choice does not yet exist. Due to this heterogeneity, the results of the above-mentioned studies are of limited comparable value.

Surveys reveal differences, however, depending on whether members of the general public or insured individuals who are mainly healthy and not required to choose a hospital were interviewed as “potential” patients [34–37], or whether respondents were patients before admission or after discharge from hospital treatment [9, 22, 27–29, 38–51].

A lot of surveys in Germany address the general population or persons with health insurance. The important criteria for their hypothetical hospital choice are: qualification of medical and nursing staff, hospital specialisation, detailed data on hygiene, interventions, outcomes and patient involvement. They quote their own outpatient care providers, information material, health insurers/sickness funds and patient counselling offices as the main sources of information [34–37]. Surveys interviewing patients find as the most important decision criteria: personal experience with hospitals, recommendations from outpatient care providers and relatives, the hospital’s reputation, distance from home and ease of access, but also aspects of the patient-caregiver relation such as degree of participation, sufficient time for conversation, or friendliness of staff, and in some health care systems the waiting time and whether a hospital has a supply contract with the patient’s health insurer. Main sources of information for patients are their own experience with a hospital, outpatient caregivers and relatives [9, 22, 27–29, 38–51].

These previous studies exploring patients’ hospital choice have mainly surveyed groups of patients undergoing elective hospital interventions with a longer period prior to the intervention, such as orthopaedic or general surgery, or cardiac or vascular surgery. There is a lack of studies analysing patient behaviour in choosing a hospital in day-to-day practice, across disciplines, type of intervention and level of care, including the time that remains to patients prior to admission. Furthermore, it is not known how many patients actually can choose their hospital. Against this background, our study explored four research questions on hospital choice from the patient perspective in the German health system:

1. How many patients decide where to go for hospital treatment?
2. How much time passes from indication for hospitalisation to admission?
3. Which sources of information on hospitals do patients use prior to hospitalisation?
4. Which criteria are of relevance to patients in choosing a hospital?

## Methods

This survey is an observational cross-sectional study, based on quantitative primary data collected in a multi-centric study via questionnaire among inpatients in Germany. The population to which the study refers comprises all patients admitted for inpatient care to German hospitals in 2012 [18]. The sample was a disproportionally stratified random sample with an average of about 50 patients admitted consecutively for inpatient care in 46 departments of 17 hospitals in a total of 15 cities and towns situated in 5 different urban and rural regions in North Rhine-Westphalia (NRW). NRW is the most populated federal state with 17.6 million citizens, i.e. 22% of Germany’s population. Hospitals were selected on the basis of regional differences and the level of care and contacted with the request to participate.

Stratification criteria are the medical discipline and the level of care. 11 medical disciplines with considerable patient intake (internal medicine, gynaecology, obstetrics, paediatrics, psychiatry, orthopaedics, neurology, urology, ENT and geriatrics) have been considered in the sample. In the year of data collection in 2012 these disciplines covered 17.1 million (91.9%) of the total of 18.6 million hospital admissions to inpatient care in Germany. Disciplines not part of the sample and covering some 8.1% of all inpatients in Germany are for example ophthalmology, dermatology, oral and maxillofacial surgery, neurosurgery, and nuclear medicine.

The stratification criterion “level of care” considers 3 care levels, defined according to number of beds: standard care (hospitals with less than 200 beds), specialist care (hospitals with 200–499 beds) and maximum care (hospitals with more than 499 beds). At the level of specialist and maximum care the sample covered two hospital departments for each discipline in different hospitals. ENT as the only exception has been considered at the maximum care level only since this discipline with lower case numbers is mainly covered by affiliated doctors at the specialist care level. The standard care level comprises internal medicine and surgery. Two hospital departments of each discipline from different hospitals formed part of the sample. In order to compare the realised study sample with all hospital patients in the study year in Germany the distribution of admissions by weekday, gender and age categories are given. The sample size was calculated to obtain a confidence interval of +/− 3% for a confidence level of 95%.

Two interviewers conducted the survey between January 2012 and March 2013. The 46 hospital departments with more than 200 ward teams in total were contacted and informed about the survey in the same pre-arranged manner. Interviewers received three training units to ensure uniform procedures in the patient survey.

The stratified sample was weighted for statistical analysis according to the distribution of all inpatients of the 11 disciplines in Germany (Table 1). Weighting was not adjusted for the level of care since the sample distribution is close to the distribution in all hospitals in Germany according to the level of care, with factors of 0.8 (standard care), 1.0 (specialist care) and 1.1 (maximum care). The software IBM SPSS Statistics Version 22 was used for statistical analysis to calculate the frequency distributions of criteria for hospital choice. Approximate 95% confidence intervals were calculated based on the standard normal distribution.

**Table 1:** Study population and participant sample

Discipline	Raw data of study population					Admission for full-time inpatient care in Germany	Weighting factor per discipline	Weighted sample	
	Total	Respondents		Non-respondents		in 2012		Respondents	
		n	% (row)	n	% (row)	n		n	% (column)
Internal medicine	351	263	74.9	88	25.1	6,731,730	2.8798	758	39.4
Surgery	295	255	86.4	40	13.6	3,892,125	1.7172	438	22.8
Gynaecology	182	164	90.1	18	9.9	836,815	0.5741	94	4.9
Obstetrics	197	173	87.8	24	12.2	783,858	0.5098	88	4.6
Paediatrics	221	197	89.1	24	10.9	899,720	0.5138	101	5.2
Psychiatry	176	102	58.0	74	42.0	819,951	0.9044	92	4.8
Orthopaedics	196	177	90.3	19	9.7	794,376	0.5049	89	4.6
Neurology	227	177	78.0	50	22.0	828,473	0.5266	93	4.8
Urology	196	174	88.8	22	11.2	739,578	0.4782	83	4.3
ENT	117	88	75.2	29	24.8	581,619	0.7436	66	3.4
Geriatrics	210	155	73.8	55	26.2	201,625	0.1464	23	1.2
Total	2368	1925	81.3	443	18.7	17,109,870		1925	100

The questionnaire employed for the survey is the revised version of a questionnaire developed for an earlier project on the same issue [47]. The items offered in the item lists for decision criteria and sources of information follow those typically presented in the cited German and international studies [9, 22, 25–28, 34–51]. The questionnaire was subjected to another pre-test using the “think aloud” method with ten hospitalised patients respectively from internal medicine, surgery, gynaecology and obstetrics in order to check the comprehensibility of items and revise where necessary. Patients may fill in the questionnaire either autonomously or assisted by an interviewer. For paediatric patients the questionnaire was adjusted to be completed by parents. The questionnaire addresses previous hospital experience, the context of admission and decision, sources of information (complete itemised list in Table 4) and decision criteria (complete itemised list in Table 5) and patients’ socio demography. The two questions on sources of information and predominant selection criteria explicitly enquire which sources and criteria the respondent actually used for the present hospital admission and which were essential in the decision. Participants were free to mark each single item that is applicable and relevant with a cross (multiple selection), but were not requested to answer each item. Additional remarks could be added on the questionnaire as free text. An English translation of the questionnaire is given as an additional file (Additional file 1).

## Results

### Sample

The study population comprised 2368 patients consecutively admitted to the 46 hospital departments of whom 1925 respondents constitute the study sample with completed questionnaires (22.0% completed alone, 78.0% together with interviewer) (Table 1). The reasons for the 18.7% non-respondents were: permanent (6.3%) or current (4.7%) physical or mental impairment, already discharged (4.0%) or termination of study (1.6%) prior to contact, questionnaire not returned (1.3%), refusal to participate (1.2%) or insufficient fluency in German (0.7%). A socio-demographic comparison revealed non-respondents as 48.6% male, which is 4% more than among respondents, and as a slightly larger proportion of the age categories over 75.

Table 2 lists participants’ information on socio-demography, previous hospital experience and admission day. Compared to all hospital patients in Germany receiving inpatient care in 2012, 2.3% more women were among respondents, less patients in the age category up to 24, and more in the age group from 50 to 79. Distribution of admissions by weekday mainly corresponded to the distribution of all hospital patients in the same year [Federal Statistical Office: statistical analysis on authors’ request, 2015]. With 47.2% the hospitalisation rate over the past twelve months was almost four times as high as among the general German population [52]. Almost two thirds have already known the hospital, and 42.7% the hospital department from a previous stay.

**Table 2:** Socio-demography, previous hospital experience and admission day

	Respondents n	Respondents %	All hospital patients in Germany 2012^b^
Socio-demography			
Females	1059	55.0	52.7
Males	866	45.0	47.3
Age in years:			
0–24	179	9.4	15.2
25–29	68	3.5	3.9
30–39	150	7.8	7.7
40–49	187	9.7	10
50–59	284	14.7	13.1
60–69	300	15.6	14.2
70–79	469	24.4	20.7
80–89	238	12.4	12.9
90 and older	48	2.5	2.4
Weekday of admission	1918		
Monday	417	21.7	20.5
Tuesday	348	18.2	18.4
Wednesday	335	17.5	17.4
Thursday	302	15.8	16.2
Friday	240	12.5	12.7
Saturday	125	6.5	6.6
Sunday	150	7.8	8.0
Number of previous inpatient stays	1921	100	
None	123	6.4	n.a.^a^
1 to 5	803	41.8	n.a.
> 5	995	51.8	n.a.
Last inpatient stay	1925	100	
Within the last 12 months	907	47.2	n.a.
More than 12 months ago	875	45.6	n.a.
Previous stay in this hospital (of *n* = 1923)	1241	64.5	n.a.
Previous stay on this hospital department (of *n* = 1925)	822	42.7	n.a.

^a^n.a. = not available

^b^[60]

### Hospital choice and time prior to admission

Responding to the question “Who decided on admission to this hospital?”, 63.0% said they decided themselves. Emergency rescue services decided in 12.4% of cases, followed by family doctors and outpatient care specialists (Table 3). Asked for the time between indication of hospitalisation and admission, 55.7% said they were admitted on the day of or one day after indication, 22.7% after 2 to 7 days, and 21.1% after more than 1 week (Table 3).

**Table 3:** Decision on hospital and time between indication of hospitalisation and admission

	Respondents n	Respondents %	95% confidence interval
Decision-maker	1918	100	
Patient	1207	63.0	60.9–65.2
Emergency rescue service	237	12.4	10.9–13.9
Family doctor	164	8.5	7.3–9.8
Specialist	144	7.5	6.3–8.7
Hospital outpatient department	62	3.2	2.4–4.0
Other	54	2.8	2.1–3.5
Relatives	50	2.6	1.9–3.3
Time prior to admission	1921	100	
Admission on same day	980	51.0	48.8–53.2
Admission on following day	91	4.7	3.8–5.6
2–7 days	436	22.7	20.8–24.6
8–28 days	298	15.5	13.9–17.1
More than 4 weeks	108	5.6	4.6–6.6
Don’t know	9	0.5	0.2–0.8

### Sources of information

Previous personal experience of the hospital was the only source of information for 44.1% of patients; one quarter did not seek information; 30.5% used at least one external source of information (Table 4), whereby half of the latter group used several such sources (multiple response). Among the 14 defined external sources of information, relatives constituted the most frequent category with 14.2%. “Relatives” in this context refer to a patient’s personal social environment and include family, in-laws and partners, friends, acquaintances and colleagues. 11.6% consulted the specialist who provided outpatient treatment, 10.4% the family doctor, 9.1% the Internet and 4.7% a hospital outpatient department. Less than 2% respectively used the remaining 9 external sources of information.

**Table 4:** Sources of information used prior to admission (multiple response)

Source of information	All respondents		
	n	% of 1925	95% confidence interval
Did not obtain information (no multiple response)	489	25.4	23.5–27.3
Only personal previous experience of hospital (no multiple response)	848	44.1	41.9–46.3
1 or more external sources of information	588	30.5	28.4–32.6
External sources of information (multiple response)			
Relatives	273	14.2	12.6–15.8
Specialist	224	11.6	10.2–13
Family doctor	200	10.4	9.0–11.8
Internet	175	9.1	7.8–10.4
Hospital outpatient department	90	4.7	3.8–5.6
Personal inspection of hospital	33	1.7	1.1–2.3
Hospital information event	28	1.5	1.0–2.0
Other sources of information	23	1.2	0.7–1.7
Information brochures	20	1.0	0.6–1.4
Daily newspapers	6	0.3	0.1–0.5
Health insurance funds	5	0.3	0.1–0.5
Patient associations	1	0.1	0.0–0.2
Support groups	1	0.1	0.0–0.2
Consumer advice services	0	0.0	

### Decision criteria

Among the group of 1207 patients who chose the hospital themselves, personal previous experience of the hospital was the most frequent main decision criterion (58.7%), and for 33.5% of respondents even the only one. 25.3% mention further criteria (Table 5). The reputation of a hospital and recommendations from their own outpatient caregivers were important criteria for approximately 30%, followed by distance from home (24.9%) and recommendations from relatives (20.9%). The next two criteria referred to a participative patient-doctor relationship, i.e. whether doctors take enough time for patients (13.6%), and whether patients were included in decisions on treatment (9.6%). All of the further 11 criteria which address more specific operationalised quality criteria were rated as important by between 6% and less than 1% respectively.

**Table 5:** Important criteria for hospital choice (multiple response) for those respondents who choose the hospital themselves

Decision criteria	Patients choosing the hospital themselves		
	n	% of 1207	95% confidence interval
Personal experience with this hospital through previous hospitalisation, of these:	709	58.7	55.9–61.5
with multiple response	404	33.5	30.8–36.2
without multiple response	305	25.3	22.8–27.8
Good hospital reputation	364	30.2	27.6–32.8
Recommended by outpatient doctors (family doctor, specialist)	358	29.7	27.1–32.3
Distance from home	301	24.9	22.5–27.3
Recommended by relatives	252	20.9	18.6–23.2
Whether physicians take enough time for patients	164	13.6	11.7–15.5
Other reasons	146	12.1	10.3–13.9
Whether patients are included in treatment decisions	116	9.6	7.9–11.3
Whether medical-technical equipment is state-of-the-art	77	6.4	5.0–7.8
Treatment success record for my type of intervention	45	3.7	2.6–4.8
Accessibility of hospital by public transport	43	3.6	2.5–4.7
Whether the hospital adheres to all rules of hygiene	37	3.1	2.1–4.1
Waiting times up to admission	35	2.9	2.0–3.8
How often the hospital performs my type of intervention	32	2.7	1.8–3.6
How other patients rate the hospital in a satisfaction survey	23	1.9	1.1–2.7
How often complications occur after the intervention I require	19	1.6	0.9–2.3
Whether the hospital adheres to medical guidelines	19	1.6	0.9–2.3
How often infections occur among patients of this hospital	16	1.3	0.7–1.9
How many patients die in the intervention I require	7	0.6	57.2–62.8

## Discussion

In the German healthcare system almost two thirds of hospital patients perceive the decision on a hospital as their own choice, across disciplines, type of intervention and care level. This means that the declared health policy objective of patient participation in the choice of a hospital is often accomplished. This high percentage has to be contextualised by the high level of choice in the German health care system where patients have and expect free choice of providers in the ambulatory setting for primary and all secondary care by specialists, and now for years in the tertiary inpatient hospital care as well. Assigned care providers, often part of tax-funded health care systems with strong gate-keeping functions, are not characteristic for the German social health care system [53].

But what about the policy objective to have patients base their choice on public quality reporting made available for this purpose? Such performance reports are now published for each German hospital on an annual basis and available via Internet. However, more than half of patients are admitted to the hospital on the day of indication or one day later, so that in effect many patients do not have sufficient time to find, review, evaluate and compare hospital quality reports and make them part of their decision. It remains to determine how much time on average patients require – either alone or assisted by relatives – to obtain the required information on the planned intervention and base their hospital choice on a comparative decision. It appears evident that hours or even a few days are not enough for a majority of patients. Assuming at least one week prior to admission as sufficient, 21% of patients would have the time to review quality reports; if a period of at least 4 weeks is defined as sufficient time to obtain information, the percentage would be 6%. Apart from participation options and the time available prior to admission as significant framework conditions for hospital choice, another result of the study is of practical relevance as well. The majority of hospital patients have previous experience with hospitals; half of them were hospitalised during the past 12 months, and almost two thirds know the hospital personally from a previous stay, more than 40% from a stay even in the same department. This explains that for many patients (44%) their personal knowledge of the hospital constitutes the immediately available and exclusively used source of information. Only one third of patients obtain information from external sources, partially in addition to their own hospital experience. The most important external sources of information are relatives and outpatient caregivers, followed by the Internet and information received from the hospital. Hence this third of all patients, who indeed address external sources, rely especially on immediate and personal access to information based on familiar, trusted third parties.

As a result, personal previous experience with the hospital is the most frequent decision criterion for more than 50% of patients who choose the hospital themselves. This means that most patients choose and stay with what they know best in the situation being confronted with a hospitalisation. Recommendations from outpatient caregivers and the hospital’s reputation are relevant criteria for almost one third, followed by distance from home, relatives’ recommendations and aspects of an attentive and participative caregiver-patient relationship at the hospital. These criteria have far more relevance for patient decisions compared to single defined quality indicators often given in quality reports, such as complication rates, hygiene indices, intervention frequency or mortality figures. As with the favoured information sources, patients prefer aggregate and evaluative information in the form of personally conveyed experience or recommendation by familiar, trusted (expert) persons.

These results confirm the findings in international studies and indicate that patients behave similarly across very diverse organised and financed health care systems, and the patients’ choice behaviour seems to be rather stable over time even though more evidence-based quality information are available in the last years in many countries [9, 22, 27–29, 38–51]. But what is not addressed in our analyses are possible patient subgroup differences according to sociodemographic patient characteristics such as gender, age, socioeconomic status, migration status, or language competency. All these factors can influence choice behaviour and even the possibility to exercise choice as shown by Fotaki [54, 55].

What do our findings on hospital choice of a representative inpatient sample indicate? Is treatment quality as described best by scientifically sound, objective, evidence based quality measures not relevant to patients in choosing a hospital as their place of treatment? Yes and no, yes, the best quality is most relevant for patients, and no, for most patients quality indicators well presented in report cards are of low practical relevance. This fact is well known in the literature [7, 15, 20, 21, 23] and addressed as a cognitive problem of comprehension by the respective consumers. In fact, it might be that it is not a consumer who decides in the situation of a personal upcoming hospitalisation, but a patient. As Marshall and McLoughlin [33] point out the patients’ specific situation when choosing a hospital must be considered to understand their preference for aggregate information based on personal recommendation. These authors refer to psychological and sociological theories to understand and model patients’ decision-making: “These theories regard decision making as primarily a social process rather than a cognitive one. People draw on past experiences and are influenced by their expectations and fears and by the views of others - particularly people they trust”. [33]. Therefore free choice of a hospital is more than the possibility to choose, the time to consider, and the availability of well adapted quality information. The important role of trust building in this situation is described by Geuter [56] in a qualitative study with hospitalised patients: “Patients require individually and emotionally compatible information referring to professional care providers which they mainly obtain from their own social network or from outpatient caregivers, and which enable them to build up trust. Trust in the persons who provide care at the hospital constitutes a core factor determining a patient’s decision-making process”.

### Limitations

The sample has some limitations. Medical disciplines not included in the study and covering 8% of admissions may affect results. But we see no evidence that these patients would behave in a basically different manner. The 19% non-respondents may distort our results to some degree; above all, the largest group (6%) of patients incapable of being interviewed on a permanent basis suggests a less active decision behaviour. The study was conducted in one German state exclusively, so that potential regional characteristics of other states remain unaccounted for. However, structures and procedures in hospital care do not differ fundamentally between German states. Possible effects have at least been partially offset by consideration of care levels and weighting adjustment according to medical disciplines on the federal level.

## Conclusions

In view of the framework conditions identified and of patients’ considerations in choosing a hospital, two conclusions appear to be of particular relevance.

First, for lack of time, or as a matter of general preference, many patients require aggregate quality information in the form of recommendations instead of personally researched quality indicators when choosing a hospital. At least at the time of the indication for hospitalisation, outpatient caregivers are always involved in the patient’s decision-making, and frequently they are the only advisors with medical competence in the decision process. This fact assigns them the core role of “agents” who need to be responsible enough to base their advice on quality information and thus help the patient to use quality reporting. This applies also to the one third of cases where patients do not choose the hospital themselves. Access to, and forms of presenting, quality data should therefore be geared not only to the needs of patients but also specifically to the needs of these professional users [57, 58]. This agent function involves a high degree of responsibility, i.e. the motivation to keep the patient’s best interest in mind and not serve one’s own interests or those of third parties.

Second, personal experience with hospital treatment is a core criterion in a patient’s subsequent decision for or against a hospital, and is therefore of primary importance. Caregivers in the hospital may consistently take this fact into account. Their efforts to create a positive treatment experience should combine subject-specific medical competence with all aspects of an attentive and participative caregiver-patient relationship [59]. Hospital treatment of this kind receives positive feedback from patients, relatives, outpatient care providers, and also in terms of the hospital’s reputation. Depending on discipline and indication a number of activities may be helpful for some patient groups to develop trust in caregivers and hospitals, such as outpatient ward consultation, informative events or guided hospital tours.

## Additional file

Additional file 1: Patient questionnaire on hospital choice in Germany. English translation of the applied patient questionnaire to survey hospital choice in Germany. (PDF 246 kb)
