# Supplementary material for: Hospital choice in Germany from the patient’s perspective: a cross-sectional study
Source: BMC Health Serv Res. 2017 Nov 13;17:720. doi: 10.1186/s12913-017-2712-3 (PMC5683328; doi:10.1186/s12913-017-2712-3)
Supplement: Additional file 1: — Patient questionnaire on hospital choice in Germany. English translation of the applied patient questionnaire to survey hospital choice in Germany. (PDF 246 kb) [file 12913_2017_2712_MOESM1_ESM.pdf]

KH: ___, FA: ___, Sub-FA: ____________, PC: ___

Questionnaire on hospital choice

1. How often have you been hospitalised (inpatient) before this hospital admission? (Regardless in which hospital.)

☐ never (0 times) ☐ 1 to 5 times ☐ more than 5 times ☐ don't know

2. When was your last inpatient stay in a hospital?

☐ it is my first inpatient stay in a hospital
☐ within the last 12 months
☐ more than 12 months ago
☐ don't know

3. What were the main reasons and decision criteria to come to choose this hospital? Please list your key points:

____

____

4. Have you been hospitalised as inpatient in this hospital before??

☐ yes ☐ no ☐ don't know

4a. If yes: Have you been as well in this hospital department before?

☐ yes ☐ no ☐ don't know

5. If you have been hospitalised as inpatient in this hospital before, how satisfied have you been with your stay back then?

☐ I can't answer this question, it is my first stay in this hospital
☐ very satisfied
☐ satisfied
☐ less satisfied
☐ not satisfied
☐ don't know

6. Have you sought for information about this hospital by relatives or friends before admission?

☐ yes ☐ no ☐ don't know

7. Do you, relatives or friends of yours work in this hospital?

☐ yes ☐ no ☐ don't know

8. How far is this hospital away from home?

8a. Please specify what type of transportation you would typically take to come to this hospital.

☐ car, motorcycle
☐ public transportation (bus, train, tram, subway) or by taxi
☐ bicycle
☐ walking
☐ don't know

8b. How long does it take to get to the hospital from home? Please give the time necessary in minutes.

________ minutes

9. Is there a hospital that could treat you with your current complaints as well and that you could reach as fast or even faster or with less effort from home?

☐ yes ☐ no ☐ don't know

9a. If yes: Why haven't you chosen that hospital instead?
Please list your key points:

____

____

10. What type of transportation do your most important relatives usually take to visit you here in the hospital?

☐ car, motorcycle
☐ public transportation (bus, train, tram, subway) or by taxi
☐ bicycle
☐ walking
☐ I do not get visitors
☐ don't know

10a. How long do your most important relatives need to get to the hospital? Please give the time necessary in minutes.

________ minutes

11. Did you have an appointment for this admission?

☐ yes ☐ no ☐ don't know

11a If yes: Who made the appointment with the hospital??

☐ I myself
☐ primary care doctor
☐ specialist doctor
☐ doctor of the hospital policlinic
☐ relative
☐ other (please state): ____

12. Who admitted you to the hospital?

☐ I myself
☐ primary care doctor (or his/her substitute)
☐ specialist doctor (or his/her substitute)
☐ primary care doctor or his/her substitute
☐ hospital policlinic
☐ emergency service
☐ other (please state): ____________________________________

13. Who decided on admission to this hospital?

☐ I decided myself
☐ primary care doctor
☐ specialist doctor
☐ hospital policlinic
☐ emergency service
☐ relative
☐ other (please state): __________________________

14. Do you – or somebody else- have chosen for this admission between two or more hospitals?

☐ yes ☐ no ☐ don't know

15. How satisfied are you with your participation in choosing the hospital for this admission?

☐ very satisfied
☐ satisfied
☐ less satisfied
☐ not satisfied
☐ don't know

16. How much time before admission day did you know that you needed an inpatient stay in a hospital?

☐ I was admitted the same day.
☐ I was admitted the following day.
☐ 2 to 7 days before admission
☐ 1 to 4 weeks before admission
☐ more than 4 weeks before admission
☐ don't know

17. About how many hospitals have you sought information before this admission?

☐ about none hospital
☐ only about this hospital
☐ about this and other hospitals
☐ only about other hospitals
☐ don't know

18. Did you have enough time before admission to inform yourself about hospitals?

☐ yes ☐ no ☐ don't know
☐ I did not have to seek information since I already knew this hospital.

19. How much time do you need round about before an admission to get information on a hospital you do not know yet in order to make your choice?

about _________days
☐ don't know

20. Where [or by whom] in fact have you sought for information on hospitals before admission to make your choice?
Please mark all items that apply. (Multiple answers possible.)

☐a I did not need to seek for information since I already knew the hospital from a previous stay.
☐b I did not seek any information before admission.
☐c from my primary care doctor
☐d from my specialist doctor
☐e in the hospital policlinic
☐f from my relatives and friends
☐g from a hospital information event
☐h from the Internet (myself or somebody else)
☐i from information brochures
☐k from daily newspapers
☐l from health insurance fund
☐m from patient associations
☐n from support groups
☐o from consumer advice services
☐p from a personal inspection of the hospital
☐q other information sources: (please state):________________________________

____

21. Please return to the last question above and circle the ONE marked item that was the most important source of information for you. (When you marked only one item you do not need to circle the most important one.)

22. Which decision criteria have been important for you to choose now this hospital?
Please mark those items that were in fact important to you in choosing this hospital. (Multiple answers possible.)

☐a None, since I did not choose the hospital.

For my admission to this hospital it was important….

☐b how my own personal experience was with this hospital through previous hospitalisation in here.
☐c whether patients are included in treatment decisions
☐d whether physicians take enough time for patients
☐e whether my relatives or friends recommended this hospital to me
☐f whether my outpatient doctors (primary care doctor or specialist) recommended this hospital to me
☐g whether the hospital has a good reputation
☐h how other patients rate the hospital in a satisfaction survey
☐i how the hospital can be accessed by public transport
☐k how far the hospital is from home
☐l how long waiting times are up to admission
☐m whether medical-technical equipment is state-of-the-art
☐n how many patients die in the intervention I require
☐o how are treatment success records for my type of intervention
☐p how often complications occur after the intervention I require
☐q whether the hospital adheres to medical guidelines
☐r how often the hospital performs my type of intervention
☐s how often infections occur among patients of this hospital
☐t whether the hospital adheres to all rules of hygiene
☐u other reasons: ____

23. If you have to inform yourself on a hospital, from whom would you like to get the information??
Please mark the items most important to you. (Multiple answers possible.)

☐a from my primary care doctor
☐b from my specialist doctor
☐c directly from the hospitals in which I am interested in
☐d from websites that compare information on hospitals
☐e from consumer advice services
☐f from health insurance fund
☐g from patient associations
☐h from support groups
☐i from my relatives, friends, or colleagues
☐k from independent information centres for patients
☐l from hospital federations
☐m from others (please state): ______________________________________

24. How often do you use the Internet on average?

☐ never
☐ less than once a month
☐ at least once a month
☐ at least once a week
☐ (almost) every day

25. With which doctor(s) have you talked before admission about the treatment details?
(Multiple answers possible.)

☐a with none
☐b primary care doctor
☐c specialist
☐d hospital policlinic
☐e other (please state): ____________________________

Before patients agree upon a treatment they can get an examination by a second doctor besides the primary care doctor or the specialist. By this they can get an extra opinion and recommendation for the planned treatment. This is called to get a second opinion or "second opinion procedure".

26. Did you get a second opinion before this hospital treatment?

☐ yes ☐ no ☐ don't know

27. Did you asked for a second opinion prior to a medical treatment before?

☐ yes ☐ no ☐ don't know

28. Did you wish for a second opinion before this hospital treatment?

☐ yes ☐ no ☐ don't know

29. Do you know how to get a second opinion?

☐ yes ☐ no ☐ don't know

30. Do you have a long lasting, chronic disease, or more? Note: Chronic diseases are long lasting illnesses needing constant treatment and controls, for example diabetes or heart diseases.

☐ yes ☐ no ☐ don't know

30a. If yes: Since when do you have the (first) chronic disease?

☐ up to 1 year ☐ since 1 to 5 years ☐ more than 5 years

31. Is your current hospital stay due to a chronic disease? (diagnostics or treatment)

☐ yes ☐ no ☐ don't know

32. Have you ever gotten the diagnosis of one or more of the following diseases by a doctor? Please mark all applicable diseases and state on the right how many years ago you got the diagnosis.

		**…years ago**
☐a	cardiovascular disease (high blood pressure, disturbed cardiac blood flow, angina pectoris, coronary, chronic heart failure, cardiac arrhythmia)	
☐b	stroke or disturbed cerebral blood flow	
☐c	chronic bronchitis, bronchial asthma	
☐d	diabetes mellitus	
☐e	cancer, malignant tumour	
☐f	arthropathy (joint wear and tear/ arthrosis, inflammatory arthropathy / rheumatoid arthritis, rheumatism)	
☐g	backache	
☐h	epilepsy, Parkinson's disease, multiple sclerosis	
☐i	depression	
☐k	anxiety disorder, alcoholic disease, schizophrenia	
☐l	other chronic disease (please state): ______________________________	

33. Do you currently take any drug <u>regularly</u> and <u>for more than 6 months</u>?

☐ yes ☐ no ☐don't know

34. Do you have any disability that is officially recognised by the office for disabled?

☐ yes ☐ no ☐don't know

34a. <u>If yes</u>: What is the current degree of disability?

___________ % (per cent) ☐ don't know

35. Do you have a loss in earning capacity (ECR) recognised by the Statutory accident Insurance?

☐ yes ☐ no ☐don't know

35a. <u>If yes</u>: What is the current degree of reduction?

___________ % (per cent) ☐ don't know

36. Do you have any need for a long-term care that is recognised by the Statutory Long-term Care Insurance?

☐ yes ☐ no ☐ don't know

If yes: 36a. What level of care do you have?

level of care: ☐ I (1) ☐ II (2) ☐ III (3) ☐ don't know

37. What is your health care insurance?

☐ statutory health care insurance
☐ statutory health care insurance AND private supplementary insurance
☐ private health insurance
☐ state benefit insurance
☐ other: (for example: no health insurance, self-payer)
☐ don't know

38. Year of birth: 19 ☐☐

39. Your sex: ☐ male ☐ female

40. Since when do you mainly live in Germany?

☐ since birth since: ☐☐☐☐ (year)

41. Do you have:

the German citizenship ☐ yes
permanent residency status ☐ yes
temporary residency status ☐ yes

42. In which country were your parents born?

mother: ☐ in Germany in another country: which ? ____________________

father: ☐ in Germany in another country: which ? ____________________

43. Is German your native language?

☐ yes ☐ no ☐ don't know

43a. If not: If German is not your native language, how do you assess your knowledge of the German language?

☐ very good ☐ good ☐ fair ☐ poor
☐ can't assess

44. What type of vocational training do you have?
Which items apply to you?
If you are still in vocational training, please state so in G or H. For pupils without vocational training, please mark A. (Multiple answers possible.)

☐ A. I have no vocational qualification and I am not currently in vocational training.
☐ B. I have completed an in-company training or vocational school.
☐ C. I completed a vocational qualification from a technical school, master school, engineering school, vocational academy or professional academy.
☐ D. I have a higher technical college qualification.
☐ E. I have a university degree.
☐ F. I have a different type of vocational qualification.
☐ G. I am still in vocational training (apprentice, trainee, in-company training/ vocational school).
☐ H. I am a student.

45. To which group belongs the profession you currently perform or performed at last?
(please fill in also when receiving pension or being unemployed)

☐ I have never been employed/working.

☐a self-employed farmer / farmer in a cooperative society (including family worker)
☐b academic in liberal profession
☐c self-employed in trade, commerce, craft, industry and other...

civil servant / judge / professional soldier...
- ☐d in lower or intermediate service
- ☐e in upper intermediate service
- ☐f in higher service

employee...
- ☐g with working activity under direction
- ☐h with independent performance in responsible position
- ☐i with extensive management responsibilities

worker, namely…
- ☐k unskilled / semi-skilled
- ☐l craftsman / craftswoman
- ☐m foreman, group leader / master, overseer, work team leader

☐n in vocational training / school-college / academic studies/ apprenticeship
☐o federal voluntary service, VSY (voluntary social year)

46. How many persons are permanently living in your household, please count yourself as well? Please include all children living in the household.

_____ children (under 18 years of age) _____ adults (all persons over 18 years of age)

47. What is the total monthly net income of your household, counted from all persons who derive income?
It is meant the sum of wages, salary, income from self-employment, pension, each after discounting taxes and social security contributions. Please count as well income from public benefit and state aid, income from letting, leasing, housing support, child benefit and other income.

☐ less than 500 Euro
☐ 500 up to 1.000 Euro
☐ 1.000 up to 1.500 Euro
☐ 1.500 up to 2.000 Euro
☐ 2.000 up to 2.500 Euro
☐ 2.500 up to 3.000 Euro
☐ 3.000 up to 4.000 Euro
☐ 4.000 up to 5.000 Euro
☐ 5.000 Euro and more

48. On what type of health issues have you looked for information within the last 12 months? (please mark all applicable items!)

☐ diseases
☐ treatments
☐ hospitals (addresses, admission procedure, waiting times, quality)
☐ doctors (addresses, waiting times, quality, specialty practices)
☐ preventive measures (for example cancer prevention, smoking cessation, change of diet)
☐ alternative medicine
☐ health care insurance
☐ legal assistance for medical malpractice
☐ other
☐ Did not look for any health issues.

Thank you very much for your participation.
